# Cytokine cascades induced by mechanical trauma injury alter voltage-gated sodium channel activity in intact cortical neurons

**DOI:** 10.1186/s12974-017-0847-0

**Published:** 2017-03-31

**Authors:** Weiqiang Chen, Jiangtao Sheng, Jingfang Guo, Guoyi Peng, Jinfang Hong, Bingbing Li, Xiaoxuan Chen, Kangsheng Li, Shousen Wang

**Affiliations:** 1 Department of Neurosurgery, Fuzhou General Hospital, Xiamen University Medical College, 156 North Road, West Second Ring, Fuzhou, 350025 Fujian China; 2 Department of Neurosurgery, First Affiliated Hospital, Shantou University Medical College, 57 Changping Road, Shantou, 515041 Guangdong China; 3 Department of Microbiology and Immunology, Key Immunopathology Laboratory of Guangdong Province, Shantou University Medical College, 22 Xinling Road, Shantou, 515041 Guangdong China

**Keywords:** Mechanical brain injury, Inflammatory microenvironment, Cytokine cascades, Voltage-gated sodium channel, Nerve excitability, Brain-derived neurotrophic factor

## Abstract

**Background:**

Traumatic brain injury (TBI) triggers both immediate (primary) and long-term (secondary) tissue damages. Secondary damages can last from hours to days or even a lifetime. Secondary damages implicate several mechanisms, including influence of inflammatory mediators, mainly cytokines, on excitability of ion channels. However, studies should further explore the effects of inflammatory cytokines on voltage-gated sodium channels (VGSCs) and excitability in distal intact neurons.

**Methods:**

Mixed cultures of mouse cortical astrocytes and neurons were subjected to mechanical injury (trauma) to mimic TBI in vitro. Expression of various cytokines in these cultures were measured by real-time polymerase chain reaction and enzyme-linked immunosorbent assay. A trauma-conditioned medium with or without brain-derived neurotrophic factor (BDNF) was added to mouse primary cortical neurons for 6 and 24 h to mimic combined effects of multiple inflammatory cytokines on VGSCs. Spike behaviors of distal intact neurons were examined by whole-cell patch-clamp recordings.

**Results:**

Mechanical injury in mixed cortical neuron–astrocyte cultures significantly increased expression levels of multiple cytokines, including interleukin (IL)-1β, IL-6, tumor necrosis factor-α, monocyte chemoattractant protein-1, chemokine (C-C motif) ligand-5, IL-10, and transforming growth factor-β1, at 6 and 24 h after injury. Incubation in trauma-conditioned medium increased functional VGSCs in neuronal membranes and Na^+^ currents. Enhanced VGSCs were almost completely abolished by BDNF, and reinforcement of Na^+^ currents was also reduced in a dose-dependent manner. BDNF (30 ng/mL) also significantly reversed reduced neuronal cell viability, which was induced by medium conditioned at 6 h. At 6 and 24 h, trauma-conditioned medium significantly increased spike frequency but not spike threshold.

**Conclusions:**

In TBI, the combined effect of inflammatory cytokines is directly involved in VGSC, Na^+^ current, and excitability dysfunction in distal intact neurons. BDNF may partly exert neuroprotective effects by maintaining balance of VGSC function in distal intact neurons.

## Background

Traumatic brain injury (TBI) is a leading cause of death and disability in young adults [1, 2]. Effective management of TBI must consider that after trauma, tissue damage comprises both primary and secondary mechanisms [3, 4]. Secondary injuries last for hours to days or even a lifetime [4]. Secondary damage can be induced by processes triggered by initial injury; examples of such processes include ischemia, increased intracranial pressure, infection, inflammation, and neurodegeneration. Neuroinflammation may also persist for months to years [5].

Neuroinflammation contributes to mechanism of secondary injury [6]. Persistent neuroinflammation may influence spread of abnormal proteins and can cause neurodegeneration following TBI [7]. Earliest inflammatory activation after tissue injury is assumed to be triggered by extravasated blood products, intracellular components, reactive oxygen, and nitrogen species. These are detected by microglia and astrocytes, which sense perturbation of tissue homeostasis [8, 9]. This is followed by excitotoxicity, oxidative stress, and apoptosis [10–12].

Multiple cytokines are necessary to maintain normal brain function and repair TBI. However, they may play a pivotal role in the pathogenesis of neuroinflammation-mediated secondary damage following TBI [6, 13]. Gene profiling has shown that multiple cytokines (e.g., interleukin (IL)-1β, IL-6, tumor necrosis factor (TNF)-α, IL-10, and transforming growth factor (TGF)-β1) are strongly upregulated during acute phase after TBI [14, 15]. Over time, increased cytokines may participate in maladaptive secondary injury reactions [6]. Consistent with this phenomenon, cytokine suppression effectively ameliorates neurological damages, such as seizure, epilepsy, Alzheimer’s disease, Parkinson’s disease, autism, and multiple sclerosis, following TBI in animal models [16]. Proinflammatory cytokines increase brain excitability and seizure susceptibility by upregulating excitatory glutamatergic transmission and downregulating inhibitory gamma-aminobutyric acid-ergic transmission [17–19].

Mild TBI (mTBI) can produce long-lasting cognitive dysfunction without damaging neurons, and functional changes in intact neurons may contribute to these symptoms [20, 21]. Network dysfunction following mTBI may be attributed to altered neuronal excitability in intact neurons. Voltage-gated sodium channels (VGSCs) are crucial ion channels for the production of action potentials and neuronal excitability [23–26]. VGSCs are involved in diffuse axonal injury following TBI in mice [27]. Mechanical trauma of axons initiates a Na^+^ influx through VGSCs and subsequently triggers Ca^2+^ influx and neuronal death [28].

The influence of damaged or dead cells on distal intact neurons needs to be further explored, especially on ion channel function and neuronal excitability in the acute phase of TBI. We have previously shown that TNF-α significantly enhances Na^+^ currents by upregulating VGSC expression and that brain-derived neurotrophic factor (BDNF) likely protects neurons from excitatory toxicity by downregulating Na^+^ currents in neurons [29, 30]. Here, we use an in vitro TBI model comprising mixed cultures of primary cortical astrocytes and neurons was used to evaluate the effects of inflammatory cytokine cascades on VGSCs and the excitability of distal intact neurons after TBI. The potential therapeutic role of BDNF in mechanical trauma injury was also examined.

## Methods

### Animal procedures

Pregnant C57 BL/6J mice were ordered from the Shantou University Medical College Experimental Animal Center, Shantou, China. Animals were conducted according to the NIH Guide for Care and Use of Laboratory Animals.

### Cortical neuron culture

Primary cultures of mouse cortical neurons were established, as previously described [29, 30], by using post-natal day 1 (P1) mice. In brief, cerebral cortices (without hippocampus) were trypsinized for 2 min with 4 mL 0.25% trypsin (Invitrogen) at 37 °C and 0.5 mL fetal bovine serum (Invitrogen). Cells were collected by centrifugation at 900*g* for 10 min, resuspended in minimum essential medium (Invitrogen), and seeded onto 12 mm × 12 mm glass cover slips (2 × 10^3^ cells/mm^2^) pretreated with 12.5 μg/mL poly-d-lysine (Sigma). Glutamine (2 mM, Sigma) and 2% B-27 supplement (Invitrogen) were added to neurobasal medium immediately before use. Cultures were incubated in 2 mL culture medium at 37 °C in 5% CO_2_ atmosphere. Half of the culture medium was changed every 3 days. At day 3, cultures were exposed for 24 h to selective replication inhibitor arabinosylcytosine C at a final concentration of 4 μM to eliminate glial cells.

### Cortical neuron–astrocyte mixed cultures and mechanical trauma injury model

Neurons and astrocytes were cultured together as previously described [31]. Briefly, cortical tissues from P1 mice were isolated and dissociated, and individual cells (2 × 10^3^ cells/mm^2^) were seeded on previously prepared confluent 2-week-old astrocyte cultures with Dulbecco’s Modified Eagle Medium (DMEM)/F12 medium containing 10% fetal calf serum (Invitrogen). After 10–12 days, cocultures were subjected to mechanical injury (trauma) to mimic TBI in vitro [22, 31]. A sterile 21-gauge needle was used to make parallel scratches across circular wells of culture plates (9 × 9 scratches in six-well plates and 6 × 6 scratches in 12-well plates). Control wells were left uninjured. Medium was replaced with serum-free DMEM/F12 medium, and cultures were incubated at 37 °C.

### Effects of trauma-conditioned medium on primary cortical cultures

At 6 or 24 h after injury, the medium conditioned by cells subjected to injury was collected and added to primary uninjured cortical neurons. Uninjured (intact) neurons were plated in 12-well plates (1 × 10^5^ cells per well), incubated for 24 h, and washed thrice with prewarmed neurobasal medium. Intact cultures were exposed for 6 or 24 h in mixed medium containing 500 μL of trauma-conditioned medium [with or without BDNF (30 ng/mL, CST)] and 500 μL of fresh neurobasal medium. Control cells were incubated in a mixed medium containing 500 μL of medium conditioned by uninjured cells and 500 μL of fresh neurobasal medium. Cells were incubated for 6 or 24 h in mixed medium for electrophysiological recording.

### Electrophysiological properties of VGSCs

Electrophysiological recording of cortical cultures was performed as previously described [29, 30]. The bath solution during whole-cell recording of voltage-gated Na^+^ currents contained 140 mM NaCl, 5 mM KCl, 1 mM MgCl_2_, 2 mM CaCl_2_, 10 mM HEPES, 4 mM TEA-Cl, 0.1 mM CdCl_2_, and 10 mM glucose. The pH was adjusted to 7.3 using NaOH. The pipette solution contained 145 mM CsCl, 1 mM MgCl_2_, 1 mM CaCl_2_, 1 mM EGTA, 10 mM HEPES, and 5 mM Na_2_ATP. The pH was adjusted to 7.3 using CsOH.

Patch pipettes were pulled from borosilicate glass capillaries to a tip resistance of 2–5 MΩ using a P-97 micropipette puller (Sutter Instruments). Voltage-clamp recording was performed using an EPC-10 amplifier (HEKA), with series resistance compensated by 70–90%, and data frequency was recorded at 200 kHz. To examine the activation of VGSCs, neurons were held at −100 mV and depolarized to 100 mV in 5 mV steps, each of which lasted 20 ms and occurred at a frequency of 0.5 Hz. To examine the inactivation of VGSCs, Na^+^ currents were recorded at 0 mV following a pre-pulse from −70 to 50 mV for 40 ms with 5 mV steps.

Action potentials were recorded in current-clamp mode to measure spike thresholds and firing rates. Cells were held at 0 pA, and potentials were elicited using 120 ms depolarizing currents that were varied stepwise from −50 to 70 pA in 10 pA steps or a ramp current of 0–500 pA lasting 100 ms. The external solution contained 140 mM NaCl, 3 mM KCl, 2 mM MgCl_2_, 0.1 mM CdCl_2_, 1 mM CaCl_2_, and 10 mM HEPES. The pH was adjusted to 7.3 using NaOH. The pipette solution contained 140 mM KCl, 10 mM EGTA, 5 mM Mg-ATP, and 5 mM HEPES. The pH was adjusted to 7.3 using KOH. All experiments were performed at 23–25 °C.

### Expression of proinflammatory cytokines

Total RNA was extracted from mouse primary mixed neuron–astrocyte cultures after mechanical injury as described above. An aliquot of total RNA (5 μg) was reverse-transcribed using oligo (dT) primers and Super-Script II reverse transcriptase (Invitrogen) in a total volume of 20 μl. Levels of messenger RNAs (mRNAs) encoding pro-inflammatory cytokines were quantified by quantitative real-time PCR (qPCR) using the primers in Table 1 and the Power SYBR Green PCR Master Mix Kit (Invitrogen) in a 7500 real-time PCR system (Applied Biosystems) according to the manufacturer’s instructions. Specificity of the SYBR Green PCR signal was confirmed by melting curve analysis. In each experiment, mouse GAPDH mRNA was amplified as an internal control.

**Table 1:** Primers used for qPCR

Gene	Forward primer	Reverse primer
mIL-1β	GTGGCTGTGGAGAAGCTGTG	GAAGGTCCACGGGAAAGACAC
mIL-6	CCAGAAACCGCTATGAAGTTCC	TTGTCACCAGCATCAGTCCC
mTNF-α	ACAGAAAGCATGATCCGCG	GCCCCCCATCTTTTGGG
mIL-10	TGCTATGCTGCCTGCTCTTA	TCATTTCCGATAAGGCTTGG
mTGF-β1	GACTCTCCACCTGCAAGACC	CGTCAAAAGACAGCCACTCA
mMCP-1	TTAAAAACCTGGATCGGAACCAA	GCATTAGCTTCAGATTTACGGGT
mCCL-5	GCTGCTTTGCCTACCTCTCC	TCGAGTGACAAACACGACTGC
mCX3CL1	ACGAAATGCGAAATCATGTGC	CTGTGTCGTCTCCAGGACAA
mCD200	CTCTCCACCTACAGCCTGATT	AGAACATCGTAAGGATGCAGTTG
mNGF	CCAGTGAAATTAGGCTCCCTG	CCTTGGCAAAACCTTTATTGGG
mBDNF	TCATACTTCGGTTGCATGAAGG	AGACCTCTCGAACCTGCCC
mTrkB.FL	AGCAATCGGGAGCATCTCT	CTGGCAGAGTCATCGTCGT
mTrkB.T1	AGCAATCGGGAGCATCTCT	TACCCATCCAGTGGGATCTT
p75NTR	CTAGGGGTGTCCTTTGGAGGT	CAGGGTTCACACACGGTCT
mGAPDH	GTGCTCTCTGCTCCTCCCTGT	CGGCCAAATCCGTTCACACCG

To complement these mRNA analyses, cytokine expression was measured in the medium of mixed cultures using commercially available enzyme-linked immunosorbent assay (ELISA) kits (R&D Systems).

### Levels of functional VGSCs in cortical neuron membranes

Membrane fractions were prepared from mouse cortical neurons treated by the conditioned medium using discontinuous sucrose gradient centrifugation. Briefly, lysate in 0.32 M sucrose/5 mM Tris (pH 7.4) was layered onto 1.2 M sucrose/5 mM Tris (pH 7.4) and centrifuged at 10,000*g* for 30 min. The layer at 0.8–1.2 M sucrose was collected, diluted twofold with 0.8 M sucrose/5 mM Tris (pH 7.4), and centrifuged at 20,000*g* for 20 min. The resulting pellet was re-suspended in a RIPA buffer containing 25 mM Tris, 150 mM NaCl, 1 mM EDTA, and 2% Triton X-100 (pH 7.4) and then centrifuged at 20,000*g* for 20 min to yield the final membrane preparation (supernatant). Complete protease inhibitor (Roche) was included throughout the procedure.

The membrane protein preparations were fractionated by SDS-PAGE (100 μg protein per well) and the gel band with pan-Nav and β-actin was cut horizontally and transferred onto a Hydrophobic PVDF membrane (Millipore) separately. The membrane was blocked with 5% non-fat milk. The pan-Nav and β-actin band were separately probed with a mouse anti-pan-Nav antibody (1:1000; Sigma) and rabbit anti-β-actin antibody (1:1000; Sigma) overnight at 4 °C. The membrane was washed with TBS/Tween-20, incubated in horseradish peroxidase-conjugated secondary antibody (goat anti-mouse 1:2000 or goat anti-mouse 1:2000 Sigma), washed again with TBS/Tween-20, and visualized by standard chemiluminescence. Ensure the signal images of pan-Nav or β-actin in every independent repeat experiments have a similar but not saturated exposure. Quantification of western blots was obtained from three separate experiments.

### Statistical analysis

Data were expressed as means ± SEM and analyzed using Origin (Origin Lab Corporation, Northampton) and SPSS 15.0 (IBM, Chicago, IL, USA) software. One-way ANOVA and subsequent Tukey’s post hoc test were used to evaluate differences in voltage of half-maximal activation or inactivation (*V*_1/2_), slope (*k*) of activation and inactivation, peak current density, standard cell viability, and threshold and firing rate for action potential. Differences in cytokine secretion tested by ELISA and normative protein expression levels were analyzed using two-way ANOVA analysis. Treatment and exposure time were considered independent variables. Student’s *t* test was performed to compare differences in mRNA relative expression of BDNF receptors of the two groups. Efficiency of target amplification and normalization of products was within 0.9–1.1 in real-time PCR. Results were calculated using the 2^−(ΔΔ*CT*)^ method. *p* value of <0.05 was considered statistically significant.

## Results

### Mechanical trauma injury upregulated multiple inflammatory cytokines

**Fig. 1:**
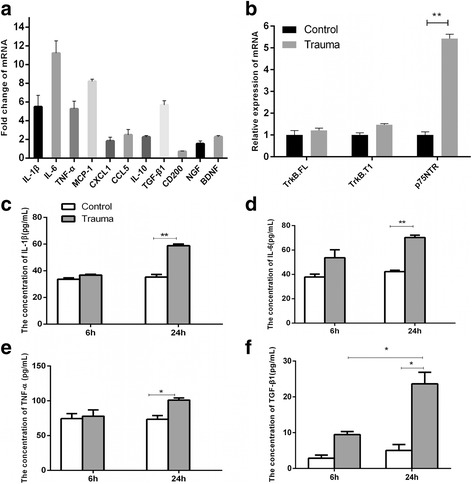
Cytokine mRNA and protein expression in medium of mechanically injured astrocyte–neuron mixed cultures. **a** mRNAs encoding IL-1β, IL-6, TNF–α, MCP-1, CCL5, IL-10, TGF-β1, CD200, NGF, and BDNF measured by real-time PCR in mechanically injured primary astrocyte–neuron mixed cultures at 6 h after injury compared with uninjured cortical mixed cultures. Values indicate mean ± SEM of three separate experiments. **b** Relative mRNA expression of BDNF high-affinity receptors TrkB.FL and TrkB.T1 and BDNF low-affinity receptor p75NTR in mechanically injured primary cortical–astrocyte–neuron mixed cultures exposed for 6 h compared with uninjured mixed cultures. Values indicate mean ± SEM of three separate experiments. **p < 0.01 (Student’s *t* test). Concentration of IL-1β (**c**), IL-6 (**d**), TNF-α (**e**), and TGF-β1 (**f**) in control medium and medium of mechanically injured mixed cultures (trauma-conditioned medium) at 6 and 24 h. Values indicate mean ± SEM of three separate experiments. *p < 0.05; **p < 0.01 (two-way ANOVA with Tukey’s post hoc test was performed with treatment and exposure time as independent variables)

Prior to testing the effect of inflammatory cytokines on VGSCs and cortical neuron excitability, we examined changes in expressions of multiple cytokines after mechanical trauma injury in neuron–astrocyte mixed cultures in vitro. As shown in Fig. 1a, mRNA expression of proinflammatory cytokines (IL-1β, IL-6, and TNF-α), chemokines (monocyte chemoattractant protein (MCP)-1, C-X-C motif chemokine ligand 1 (CXCL1), and chemokine (C-C motif) ligand 5) (CCL5)), anti-inflammatory cytokines (IL-10, TGF-β1, and OX-2 membrane glycoprotein (CD200)), and neurotrophic factors (nerve growth factor (NGF) and BDNF) increased in different degrees 6 h after trauma. IL-1β, IL-6, TNF-α, MCP-1, and TGF-β1 showed significantly higher upregulations than those of controls. ELISA was performed to determine protein levels of IL-1β, IL-6, TNF-α, and TGF-β1. Two-way ANOVA examined the effects of mechanically injured mixture and exposure duration on secretion of these proinflammatory cytokines. Mechanical injury exerted statistically significant enhancement effect on secretion concentration of IL-β1 (*F* (1, 8) = 20.27; *p* = 0.002), IL-6 (*F* (1, 8) = 16.89; *p* = 0.003), TNF-α (*F* (1, 8) = 10.33; *p* = 0.012), and TGF-β1 (*F* (1, 12) = 9.773; *p* = 0.009) in mixed glia. Exposure time significantly affected TGF-1β concentration (*F* (1, 12) = 8.126; *p* = 0.031). Tukey’s post hoc test revealed that mechanical injury significantly induced upregulation of IL-β1 (*p* = 0.008), IL-6 (*p* = 0.005), TNF-α (*p* = 0.014), and TGF-β1 (*p* = 0.041) compared with that of respective control group at 24 h (Fig. 1c–f, respectively). IL-6 and TGF-β1 increased at 6 h (Figs. 1b, d, respectively). We also examined BDNF/TrkB/p75NTR signal because BDNF/TrkB performs a neuroprotective role in various central nervous system diseases. In acute mechanical trauma injury, BDNF high-affinity receptors, namely, TrkB full-length (TrkB.FL) (*p* = 0.141) and TrkB.truncated type 1 (TrkB.T1) (*p* = 0.077), were not altered, but low-affinity receptor p75NTR (*p* = 0.036) significantly increased by fivefold at mRNA level (Fig. 1b). These results demonstrated that acute mechanical trauma injury in mixed astrocyte–neuron cultures upregulated multiple inflammatory mediators, mainly cytokines, but not neurotrophic factors in in vitro cell model of TBI.

### Exposure to trauma-conditioned medium increased Na^+^ currents in cortical neurons

**Fig. 2:**
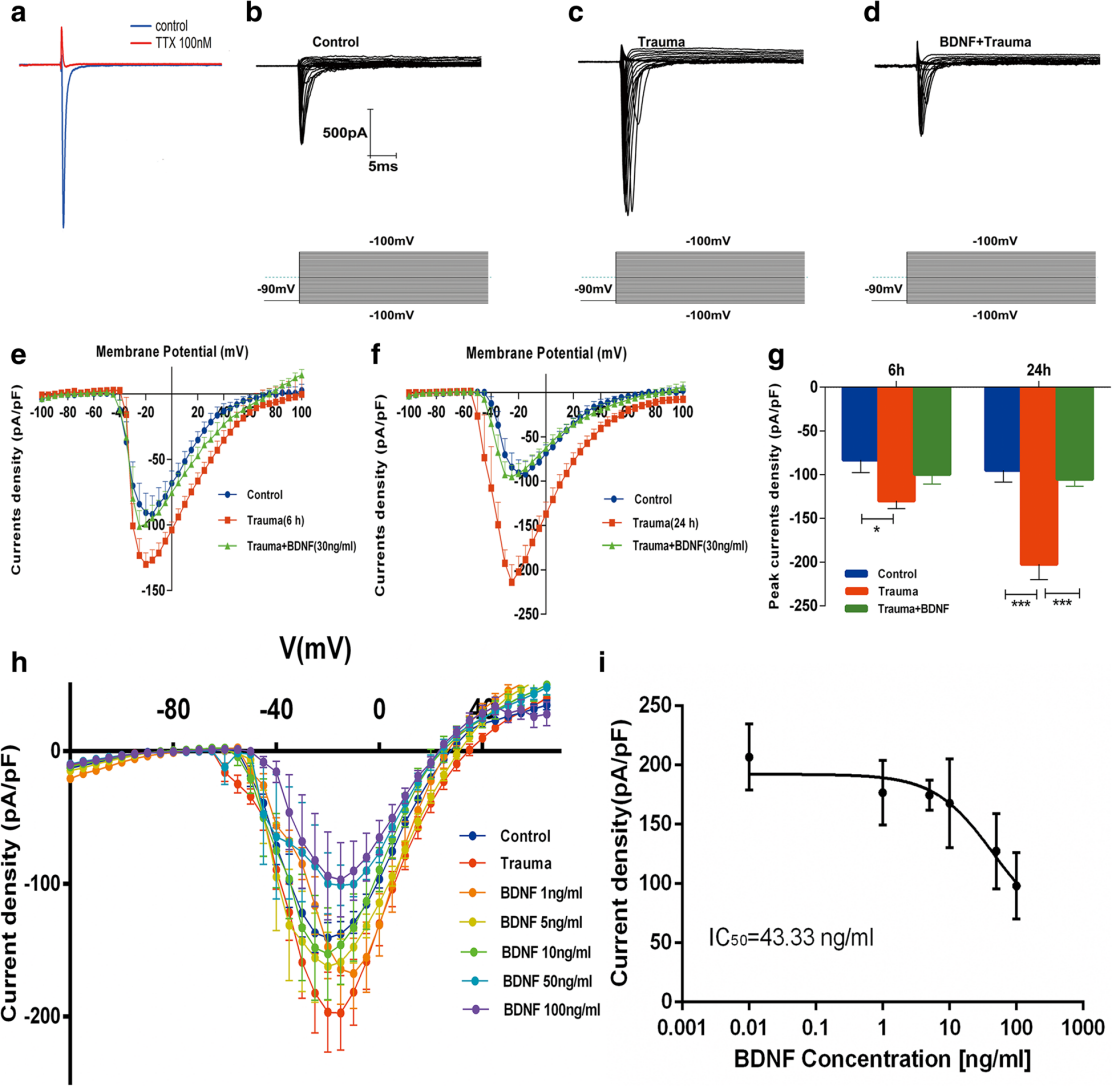
Exposure to trauma-conditioned medium increases density of Na^+^ currents. **a** Representative recording of whole-cell currents in presence or absence of TTX (100 nM). **b**–**d** Recording of whole-cell Na^+^ currents from −100 to 100 mV in untreated control neurons and neurons exposed to medium conditioned for 24 h (with or without BDNF supplementation). **e** and **f** Relationship of current density and voltage in control and trauma-conditioned medium for 6 or 24 h treatment [with or without BDNF (30 ng/ml)]. **g** Changes in peak of current density are shown in control neurons and neurons exposed to conditioned medium for 6 or 24 h by injured mixed cultures (with or without BDNF supplementation). Values indicate mean ± SEM of three separate experiments. *p < 0.05; **p < 0.01 (one-way ANOVA with Tukey’s post hoc test). **h** Current–density–voltage relationship in control exposed to conditioned medium for 24 h and exposed to conditioned medium for 24 h with BDNF (1, 5, 10, 50, and 100 ng/mL). **i** Concentration-dependent effects of BDNF (0.01–100 ng/mL) on Na^+^ current density (IC_50_ = 43.33 ng/mL)

An inward current with fast activation and inactivation was elicited by depolarization steps at −100 mV of holding potential. Such current was recorded at 200 kHz of sampling frequency in whole-cell patch-clamp recording and blocked completely using tetrodotoxin (TTX, 100 nM) (Fig. 2a). This result indicated that the currents were TTX-sensitive Na^+^ currents through VGSCs. Afterward, we examined the effect of trauma-conditioned medium containing multiple cytokines, which were secreted by mixed cultures, on voltage-gated Na^+^ current densities at 6 and 24 h. A statistically significant difference existed between groups, as determined by one-way ANOVA (*F* (2, 17) = 4.230; *p* = 0.032). Tukey’s post hoc test revealed that voltage-gated Na^+^ current densities significantly increased by 37.6% ± 4.5% in pure neuronal cultures exposed to conditioned medium harvested at 6 h after mechanical injury compared with those in control medium(*p* = 0.034) (Figs. 2b, c, e). Similarly, one-way ANOVA also showed statistically significant difference between groups at 24 h (*F* (2, 19) = 19.81; *p* <0.001). Tukey’s post hoc test also revealed that Na^+^ current densities significantly increased by 82.2% ± 5.4% in cultures exposed to trauma-conditioned medium for 24 h compared with those of controls (*p* < 0.001) (Fig. 2f). Adding BDNF (30 ng/mL) to conditioned medium almost completely abolished increased density of Na^+^ currents compared with those of controls (*p* < 0.001) (Figs. 2b–g). BDNF also showed an apparent dose-dependent effect on Na^+^ current density reduction of Na^+^ currents induced by the trauma-conditioned mediums (Fig. 2h, i). BDNF concentration appeared to correlate negatively with Na^+^ current density induced by trauma-conditioned medium in primary cortical neurons.

### Effects of trauma-conditioned medium on VGSC kinetics

**Fig. 3:**
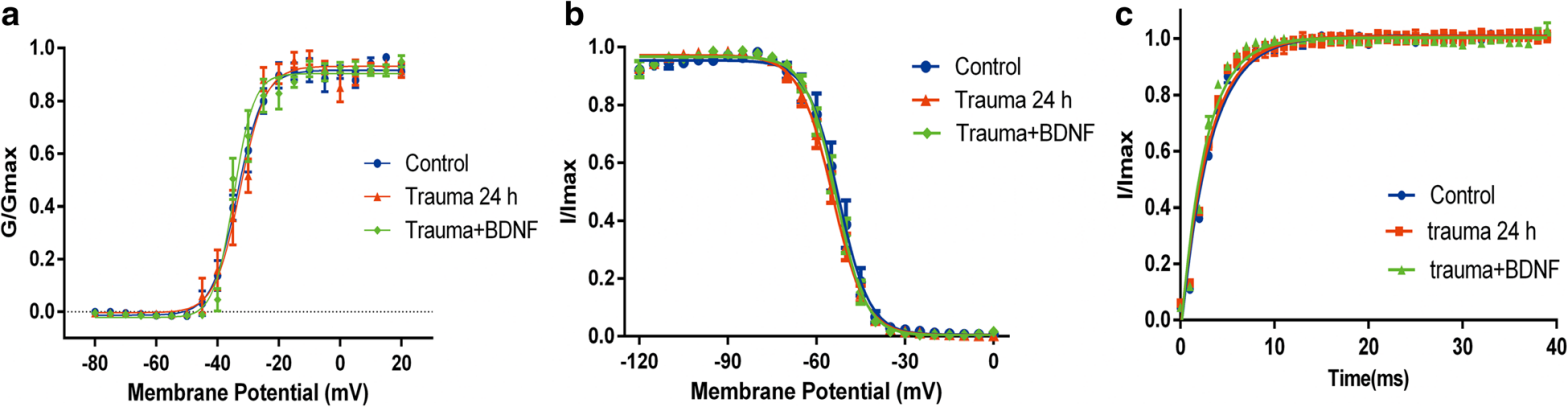
Effects of trauma-conditioned medium and BDNF on VGSC kinetics. **a** Voltage-dependent activation of VGSC in intact primary cortical neurons exposed for 24 h to trauma-conditioned medium (with or without BDNF) or in untreated neurons. **b** Voltage-dependent fast inactivation of VGSC in intact primary cortical neurons exposed for 24 h to trauma-conditioned medium (with or without BDNF) or in untreated neurons. **c** Rates of voltage-gated sodium channel recovery from fast inactivation in intact primary cortical neurons exposed to trauma-conditioned medium (with or without BDNF) or in control neurons

Conductance–voltage curves were generated and fitted using Boltzmann equation to examine activation and inactivation properties of VGSCs (Fig. 3a, b). Fast inactivation was measured by applying a peak current at 0 mV test voltage and normalizing obtained currents to maximal current (Fig. 3b). Normative currents were plotted against prepulse voltage, and current–voltage curves were fitted using the Boltzmann equation.

V_*1/2*_ of fast inactivation showed statistically significant difference between groups, as determined by one-way ANOVA (*F* (2, 23) = 8.078; *p* = 0.002). Post hoc Tukey’s test revealed that exposure of intact cortical neurons to trauma-conditioned medium did not affect VGSC activation (*p* = 0.086) (Fig. 3a), but a slight shift was observed in V_*1/2*_ of fast inactivation to hyperpolarization (from −52.47 ± 0.47 mV to −54.56 ± 0.32 mV) (*p* = 0.004) (Fig. 3b and Table 2). Similarly, a statistically significant difference was noted in V_*1/2*_ of activation between groups, as determined by one-way ANOVA [*F* (2, 23) = 4.138; *p* = 0.029]. Tukey’s post hoc test revealed that addition of BDNF (30 ng/mL) to trauma-conditioned medium slightly shifted V_*1/2*_ of activation to hyperpolarization (from −32.63 ± 0.57 mV to 34.70 ± 0.44 mV) (*p* = 0.031) (Fig. 3a and Table 2). Neither trauma-conditioned medium nor BDNF changed recovery properties of VGSCs from fast inactivation (Fig. 3c). These results suggested that both trauma-conditioned medium and BDNF did not apparently alter dynamic properties of VGSCs.

**Table 2:** Effects of trauma-conditioned media and addition of BDNF on Na^+^ channel kinetics in neurons

	Activation			Inactivation		
	Control (*n* = 9)	Trauma (*n* = 9)	Trauma + BDNF (*n* = 8)	Control (*n* = 8)	Trauma (*n* = 10)	Trauma + BDNF (*n* = 8)
*V*_*1/2*_ (mV)	−33.45 ± 0.48	−32.63 ± 0.57	−34.70 ± 0.44^#^	−52.47 ± 0.47	−54.56 ± 0.32**	−53.40 ± 0.34
*k*	4.19 ± 0.41	4.18 ± 0.50	3.29 ± 0.40	5.26 ± 0.47	5.60 ± 0.28	5.07 ± 0.29

Trauma (24 h) and trauma + BDNF (24 h). Values indicate mean ± SEM

*V*_1/2_ voltage of half-maximal activation or inactivation, *k* slope factor

**p < 0. 01 versus control; ^#^ *p* < *0.05* versus trauma (24 h) group (one-way ANOVA with Tukey’s post hoc test)

### Trauma-conditioned medium increased VGSCs in neuronal membranes and decreased survival of neurons

**Fig. 4:**
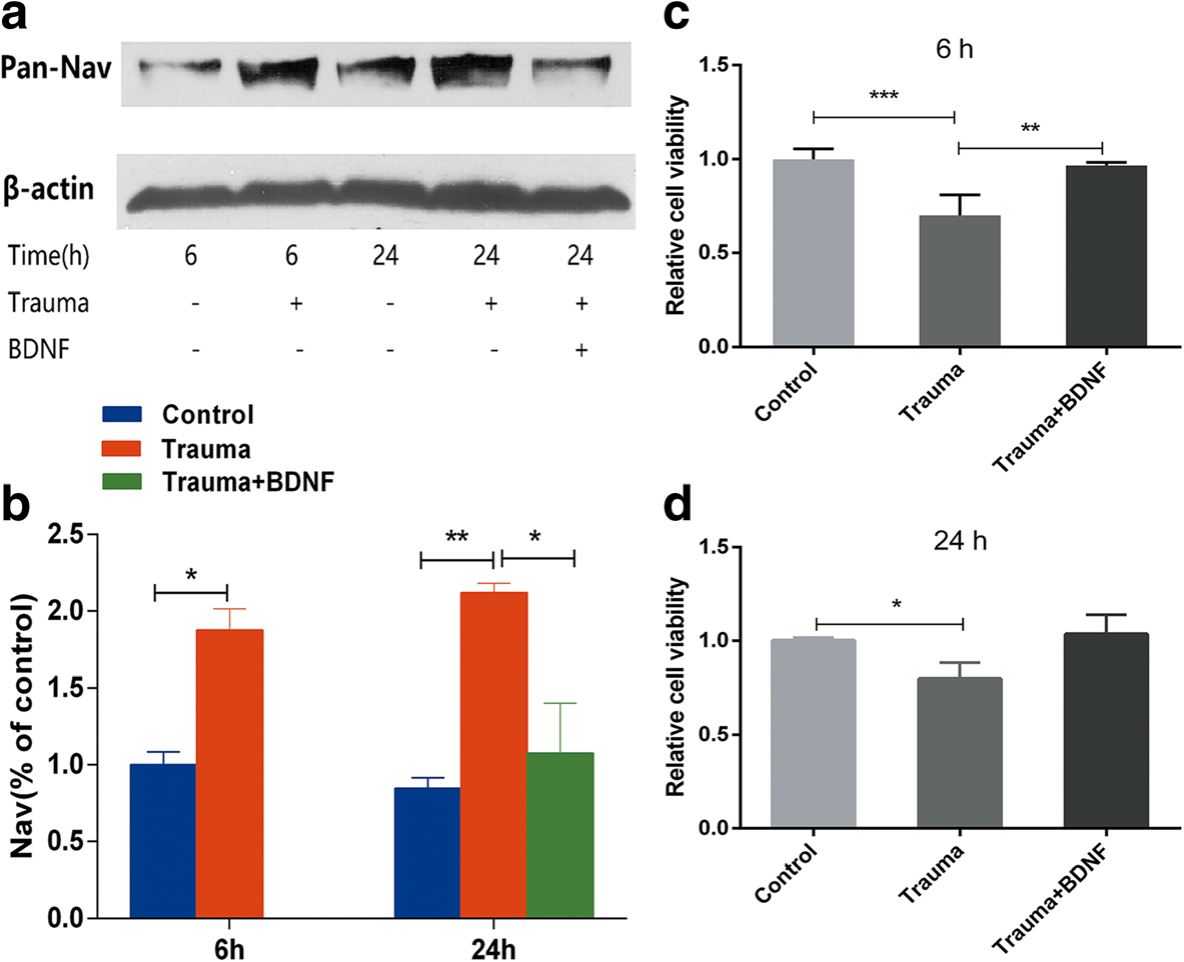
Exposure to trauma-conditioned medium increases VGSCs in membrane and decreases neuronal survival. **a** Representative immunoblotting results of VGSC expression in primary cortical neurons after exposure to conditioned medium for 6 or 24 h by injured mixed cultures (with or without BDNF supplementation). **b** Relative quantitation analysis of effects of exposure to trauma-conditioned medium on the number of VGSCs in membranes. *p < 0.05: trauma versus control; trauma + BDNF versus trauma (two-way ANOVA analysis with Tukey’s post hoc test was performed with treatment and exposure time as independent variables). **c, d** Cell viability of intact neurons in control group or exposure to conditioned medium for 6 or 24 h by injured mixed cultures (with or without BDNF supplementation) was tested by CCK-8 kit. Values indicate mean ± SEM of three separate experiments. *p < 0.05 (one-way ANOVA with Tukey’s post hoc test)

Consistent with our whole-cell patch-clamp findings, two-way ANOVA analysis showed that conditioned medium resulted in statistically significant influence on expression of functional VGSCs in plasma membrane between groups [*F* (2, 6) = 25.66; *p* = 0.002] but not on exposure time [*F* (2, 6) = 2.708; *p* = 0.151]. Post hoc Tukey’s test revealed significant increase in the number of functional VGSCs in plasma membrane (by 77.54 and 111.96%) after treatment with trauma-conditioned medium for 6 h (*p* = 0.005) and 24 (*p* = 0.048) compared with those of controls. Addition of BDNF (30 ng/mL) for 24 h almost completely reversed upregulation of VGSCs by trauma-conditioned medium (*p* = 0.037) (Fig. 4a, b). Cell viability assays were performed to determine the effect of conditioned medium on survival of intact neurons and neuroprotective effect of BDNF. Conditioned medium significantly affected cell viability of neurons at 6 [*F* (2, 15) = 15.62; *p* < 0.001] and 24 h [*F* (2, 6) = 7.896; *p* = 0.021], respectively, as determined by one-way ANOVA. Tukey’s post hoc test revealed that treatment with conditioned medium significantly decreased viability of intact neurons about 30% both at 6 (*p* < 0.001) and 24 h (*p* = 0.022). BDNF (30 ng/mL) only showed apparent neuroprotective effects on neuronal viability at 6 h (*p* = 0.006) but not at 24 h (*p* = 0.053). Altogether, these results demonstrated that upregulation of Na^+^ current density possibly resulted from increased functional VGSCs in intact neurons. Possibly, BDNF partly played its neuroprotective role by maintaining VGSCs at normal levels, thereby maintaining normal influx of Na^+^ into neurons.

### Exposure to trauma-conditioned medium altered spike behavior

**Fig. 5:**
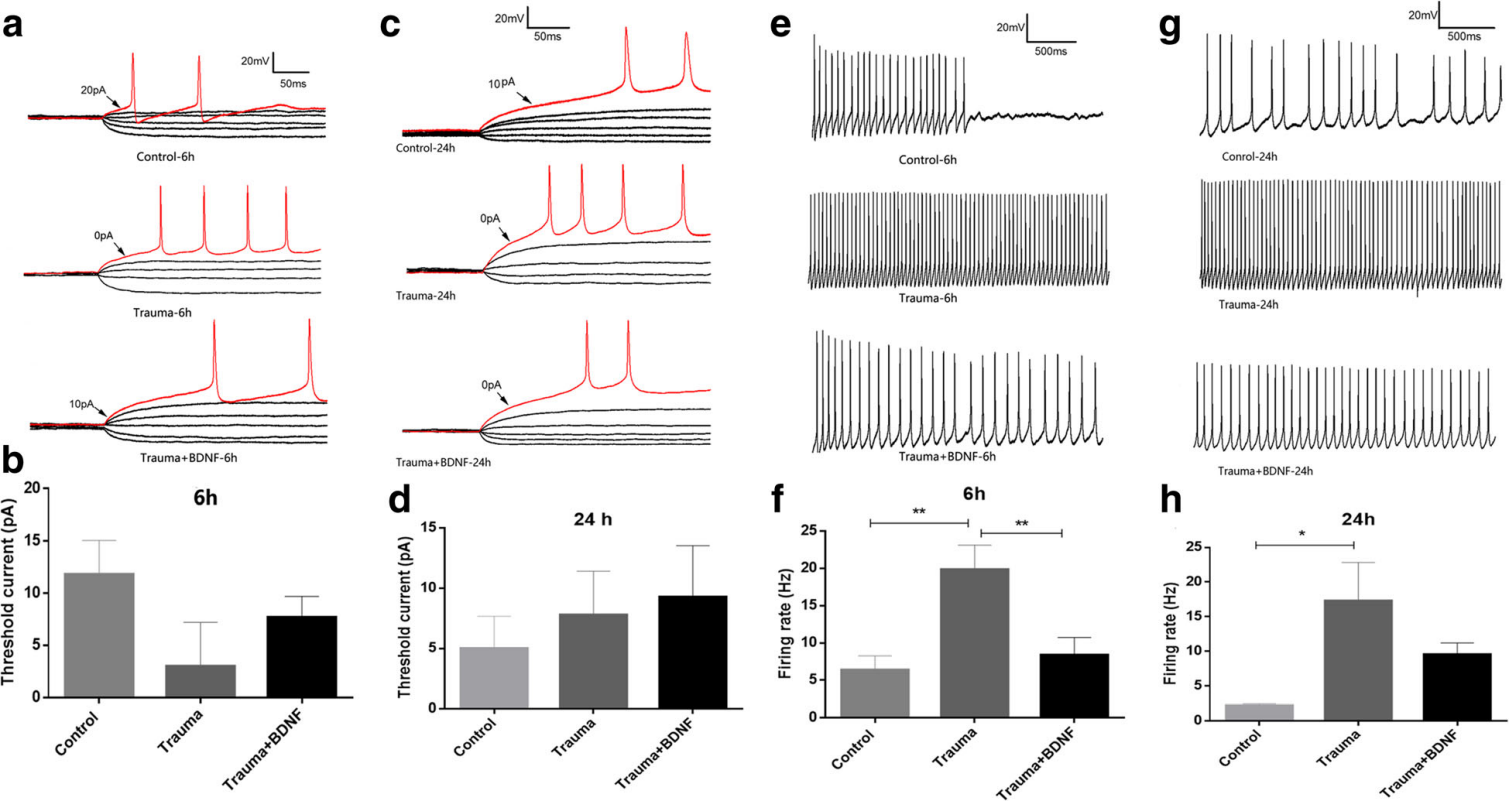
Exposure to trauma-conditioned medium alters action potentials. Representative recordings of action potentials in response to current injections in control neurons and neurons exposed for 24 h to trauma-conditioned medium (with or without BDNF) for 6 (**a**) or 24 h (**c**). Quantitation of effects of exposure to trauma-conditioned medium after 6 (**b**) or 24 h (**d**) on action potential threshold. Representative recordings of action potential firing rates in response to current injection (70 to 50 mV for 40 ms with 5 mV steps) in control neurons and neurons exposed to trauma-conditioned medium (with or without BDNF) for 6 (**e**) or 24 h (**g**). Quantitation of effects of exposure to trauma-conditioned medium after 6 (**f**) or 24 h (**h**) on firing rate. *n* = 8–10 cells per group. Values indicate mean ± SEM. *p < 0.05; **p < 0.01 (one-way ANOVA with Tukey’s post hoc test)

Examinations were performed to determine the effects of conditioned medium on spike behavior. Firing threshold of spike was not significantly altered by trauma-conditioned medium (with or without BDNF) after 6 [*F* (2, 31) = 1.855; *p* = 0.174] or 24 h [*F* (2, 28) = 0.282; *p* = 0.757] compared with respective control group (Fig. 5a–d), as determined by one-way ANOVA. Trauma-conditioned medium (with or without BDNF) significantly altered firing rate of spike at 6 [*F* (2, 32) = 7.782; *p* = 0.002] or 24 h [*F* (2, 20) = 5.826; *p* = 0.011] compared with each control group (Fig. 5e–h). Tukey’s post hoc test revealed that exposure to trauma-conditioned medium conditioned for 6 (*p* = 0.003) or 24 h (*p* = 0.013) significantly increased firing rate (Fig. 5e–h), and BDNF significantly inhibited increase in firing rate by trauma-conditioned medium conditioned for 6 h (*p* = 0.006) (Fig. 5e, f) but not at 24 h (*p* = 0.281) (Fig. 5g, h). These results indicate that the enhancement of firing rate is induced in intact neurons by the trauma-conditioned medium and that BDNF may be able to protect against the enhancement of firing rate.

## Discussion

This study demonstrated that multiple cytokine cascades were induced by mechanical injury and significantly increased VGSCs, Na^+^ currents, and excitability in intact primary cortical neurons. These findings directly implicate VGSC dysfunction in intact cortical neurons in later stages of TBI-related inflammatory microenvironment and presence of multiple pro-inflammatory cytokines. BDNF largely eliminated effects of trauma-conditioned media by inhibiting enhancement of functional membrane VGSC expression. This finding suggests that BDNF partly exerted neuroprotective effects on ion channel functions and neuronal excitability by regulating VGSC expression.

Effective management and treatment of TBI depends on short- and intermediate-term pathological assessment after injury. The majority of trauma-induced fatalities occur immediately after trauma, and a smaller number occur within 24 h of injury, which is usually caused by severe head trauma [32, 33, 34]. Trauma patients who survived the first 24 h present increased risks of immunological dysfunction and inflammatory pathology [35]. This inflammatory pathology results from increase in pro-inflammatory cytokines IL-1β, IL-6, and TNF-α [36, 37]. More accurately, multiple cytokines are released in inflammatory microenvironment of acute phase TBI [16]. Increased production of multiple cytokines was correlated with poor prognosis in TBI patients [38, 39], and cytokine suppression represents an effective method for ameliorating secondary injury after TBI [16]. Our results showed that mechanical traumatic injury not only upregulates all three pro-inflammatory cytokines at both mRNA and protein levels early after injury but also increases expressions of chemokine MCP-1 and anti-inflammatory cytokine IL-10 and TGF-β1. These results are consistent with findings of previous clinical studies [38–40]. Therefore, our mixed astrocyte–neuron culture model simulated inflammatory microenvironment of acute TBI.

Observed increase in Na^+^ currents may cause cell toxicity through several mechanisms. One potential mechanism is that augmented Na^+^ currents can increase energy required to maintain Na^+^ gradient across plasma membranes [26], leading to concurrent ATP deficiency and oxidant stress. Augmented Na^+^ currents can also activate sodium–calcium pumps, thereby increasing Ca^2+^ influx [27, 28]. Elevated Ca^2+^ levels induce excessive release of glutamic acid from presynaptic membrane, resulting in local or more far-reaching excitotoxicity and neuronal death in central nervous system [41]. Excitotoxicity participates in numerous brain diseases and injuries [42, 43]. Our results showed that augmented Na^+^ currents in healthy tissues exposed to injury-inflammatory microenvironment concurs with possible excitotoxic mechanism of secondary injury after TBI.

Multiple cytokines take part in VGSC expression through various pathways. Our study and other previous works showed that TNF-α and IL-1β enhanced VGSC currents by upregulating TTX-sensitive VGSC via p38 MAPK pathway in primary cortical neurons [29, 44]. Both CXCL13/CXCR5 and CXCL12 upregulated TTX-resistant Nav1.8 in primary sensory neurons via p38 MAPK and in primary nociceptive neurons via ERK [45, 46]. Oppositely, anti-inflammatory IL-10 can decrease VGSC current by downregulating TTX-resistant Nav1.8 expression in dorsal root ganglion neurons [47]. In the present study, we focused on combined effects of multiple cytokines on VGSCs of intact neurons in inflammatory microenvironment. Thus, enhancement in VGSC Na^+^ currents induced by conditioned medium possibly results from combined effects of multiple cytokines via various pathways; these observations still require further exploration.

We also showed that among the three types of BDNF receptors, only low-affinity p75NTR significantly increases after mechanical trauma injury; this finding is similar to those of previous studies [48–50]. p75NTR is highly expressed in developing brain during synaptogenesis and is downregulated in adult brain unless the brain is injured, in which case, it is re-expressed [51]. TrkB and P75NTR form a receptor complex for mature BDNF, and p75NTR acts indirectly to increase the number of high-affinity binding sites for TrkB [52, 53]. At least, our results indicated that BDNF may partly exert neuroprotective effects by dose dependently reversing enhancement of Na^+^ currents in secondary damage of TBI model. These findings may help in elucidating how inflammatory mediators influence electrophysiological behavior and effect of BDNF on ion channels and neuronal excitability after TBI.

Several limitations of this study should be considered. First, application of conditioned medium was lacking in lasting secretion of soluble inflammatory mediators, thus poorly simulating long duration persistence of inflammatory microenvironment in brain after TBI. Second, tests did not determine protective effects of BDNF on VGSC function of injured neurons. As shown in Fig. 2h, difference in reversal potential possibly resulted from technical problems and limited cell number for recording in each group. Difference in reversal potential disappeared with increasing number of cells for recording in each group (data not shown here). Finally, further studies should be conducted to determine whether and how inflammatory mediators directly contribute to electrophysiological behavior of non-damaged brain tissues and protective roles of BDNF by regulating VGSC function in TBI animal models.

## Conclusions

Using an in vitro model, we provide evidence that mechanical injury acts through multiple cytokine cascades to significantly modify membrane expression and electrophysiological properties of VGSCs. Injured cultures generated multiple cytokine cascades, which upregulated VGSCs, Na^+^ currents, and spike frequency. BDNF participates in neuroprotection and can reverse these effects. Our findings indicated that injured tissues or cells elicit profound effects on VGSCs and excitability of distal intact neurons through inflammatory mediators. BDNF may partly exert neuroprotective effects by maintaining the balance of VGSC function in distal intact neuron.

## Abbreviations

BDNF: Brain-derived neurotrophic factor
CCL5(RANTES): Chemokine (C-C motif) ligand 5
CXCL12: C-X-C motif chemokine 12
CXCL13: C-X-C motif chemokine 13
CXCR5: C-X-C chemokine receptor type 5
IL-10: Interleukin 10
IL-1β: Interleukin 1β
IL-6: Interleukin 6
MCP-1(CCL2): Monocyte chemoattractant protein 1
NGF: Nerve growth factor
TBI: Traumatic brain injury
TGF-β1: Transforming growth factor β1
TNF-α: Tumor necrosis factor α
VGSC: Voltage-gated sodium channel
